# Characteristics of Nonallergic Vasomotor Rhinitis

**DOI:** 10.1097/WOX.0b013e3181a8e389

**Published:** 2009-06-15

**Authors:** Jonathan A Bernstein

**Affiliations:** 1 University of Cincinnati College of Medicine, 231 Albert Sabin Way ML#563, Cincinnati, Ohio

**Keywords:** irritant triggers, nonallergic rhinitis, questionnaire, Irritant Index Scale, chronic rhinitis subtypes, survey, nonallergic vasosmotor rhinitis, vasomotor rhinitis, idiopathic rhinitis, nonallergic rhinopathy

## Abstract

Nonallergic rhinitis (NAR) conditions are currently considered diagnoses by exclusion. A diagnosis of NAR requires negative specific IgE responses by skin or serologic testing and more recently testing to exclude localized production of specific IgE in the nose. Symptoms are classically aggravated by irritant triggers such as tobacco smoke, perfumes/fragrances, and temperature or barometric pressure changes. A previously developed questionnaire survey designed to help physicians recognize differences between allergic rhinitis and nonallergic rhinitis subtypes found that patients with symptom onset later in life (> 35 years), no family history of allergies, no seasonality or cat-induced symptoms, and symptoms induced by perfumes and fragrances had > 95% likelihood of having a physician diagnosis of NAR. Of note, clinical symptoms were not generally useful for differentiating chronic rhinitis subtypes which has also been confirmed in a more recent study investigating the relationship between headaches and chronic rhinitis subtypes (Table 1). In subsequent studies it was found that a significant percentage of NAR patients did not experience irritant-induced symptoms, suggesting that these triggers are not a clinical characteristic that can be uniformly used for all NAR patients. However, a newly developed Irritant Index Scale can be used to reliably differentiate pure allergic rhinitis from nonallergic rhinitis with trigger phenotypes. The use of standardized and validated questionnaires allows objective characterization of chronic rhinitis subtypes that appears to improve the accuracy of clinically diagnosing these patients.

## Introduction

Nonallergic rhinitis (NAR) conditions are currently considered diagnoses by exclusion. To establish a definitive diagnosis of vasomotor rhinitis (VMR) and nonallergic rhinitis with eosinophilia syndrome (NARES), all other nonallergic rhinitis syndromes should be properly considered and excluded [1]. A diagnosis of NAR requires negative specific IgE responses by skin, serologic, or entopy testing. Furthermore, symptoms are classically aggravated by irritant triggers such as tobacco smoke, perfumes/fragrances, and temperature or barometric pressure changes [1]. Differentiation of the nonallergic conditions, VMR and NARES, is limited to the presence or absence of eosinophils in the nasal passages[1]. Therefore, VMR is truly a noninflammatory, nonallergic condition whereas NARES is an inflammatory, nonallergic condition. It should be emphasized that symptoms and physical findings are not pathognomonic for allergic rhinitis (AR) as patients with NAR often manifest similar features [2,3]. Therefore, proper diagnostic testing is essential to accurately classify these disorders.

## Questionnaire diagnosis of nonallergic rhinitis

An accurate diagnosis of a chronic rhinitis (CR) subtype is essential for making correct recommendations for treatment and preventing complications such as sinusitis and otitis media [1,2]. Previously, we developed a questionnaire survey designed to help physicians recognize differences between AR and NAR subtypes [3,4]. This study found patients who developed symptoms later in life (older than 35 years) and who had no family history of allergies, no seasonal allergy symptoms or perennial symptoms induced by cats or other furry pets, and symptoms induced by perfumes and fragrances had > 95% likelihood of having a physician diagnosis of NAR [3]. Of note, clinical symptoms were not generally useful for differentiating chronic rhinitis subtypes (Table 1) [3]. However, this questionnaire was less effective at accurately identifying patients with an AR phenotype [3].

**Table 1:** Prevalence of Symptoms in Patients Characterized as Allergic Rhinitis and Nonallergic Rhinitis From the Cincinnati Headache/Rhinitis Database

	Allergic Rhinitis* (n = 496)	Nonallergic Rhinitis (n = 138)	
Symptom	N (%)	N (%)	*P*
Stuffy nose	432 (87)	115 (83)	NS
Postnasal drainage	411 (83)	107 (78)	NS
Rhinorrhea	396 (80)	79 (57)	< 0.01

*Allergic rhinitis includes seasonal and perennial allergic rhinitis.

### Use of a rhinitis questionnaire to differentiate chronic rhinitis subtypes

In an attempt to see if this questionnaire would perform better if administered to a well-characterized seasonal AR (SAR) population, a modified version of the rhinitis questionnaire was distributed to 136 subjects during visit 1 of a multicenter mountain cedar SAR study [5]. The inclusion criteria for this study were having a history of SAR for 2 or more years and a positive skin prick test to Texas mountain cedar pollen that correlated with symptoms in the past year. Statistical analyses included factor analysis, hypothesis testing, and univariate regression analyses [5]. Informed consent was obtained, and the study met the ethical standards of human research according to the Helsinki Declaration of 1975 as revised in 2000.

Not surprising, symptoms were triggered in 85% (116/136) of patients while outdoors during the spring, summer, or fall; 45% (61/136) by cats; 29% (39/136) by dogs and; 21% (29/136) by furry pets. Interestingly, 54% (74/136) of patients had symptoms triggered by cigarette smoke; 72% (98/136) by weather changes; 49% (67/136) by perfumes; 35% (47/136) by incense; and 40% (54/136) by cleaning products. Thus, the majority of patients enrolled in this SAR study also experienced irritant-induced symptoms in response to perfumes, cleaning products, incense, and smoke, suggesting that they also met diagnostic criteria for a nonallergic component to their chronic rhinitis diagnosis (ie, mixed rhinitis) [5]. As previously demonstrated in developing the original questionnaire, factor analysis indicated symptoms to cat, dog, and other furry pet exposure and perfume, incense, and cleaning product exposure correlated with the Cronbach *α *for each factor being 0.78 and 0.7, respectively (≥ 0.7 reliable) [3-5].

Subsequently, the rhinitis questionnaire was distributed to subjects in 2 additional AR studies at baseline. Interestingly, over 88% of enrolled subjects in both studies indicated that they experienced significant symptoms in response to a spectrum of irritant triggers (see Tables 3 and 4, unpublished data). Therefore, the majority of patients enrolled in these SAR studies that also experienced irritant-induced symptoms in response to perfumes, cleaning products, incense, and smoke met diagnostic criteria for mixed rhinitis (MR).^2 ^These results indicate that the current criteria used to define the SAR phenotype may be inadequate. Misclassification of AR patients could explain the large number of partial or nonresponders to medication in clinical trials, thereby necessitating enrollment of a larger study population to demonstrate drug efficacy.

**Table 2:** Percentages (No.Positive Responses) of Patients With Symptoms Induced by Allergic and Nonallergic Triggers That Exhibited a "Big Effect" Indicating Extreme Satisfaction With Their Medication[5]

Drug	Cat	Dog	Furry Pets	Outdoor	Weather Changes	Tobacco Smoke	Perfume	Incense	Cleaning Agents
Oral AH	52 (15/29)	41 (12/29)	35 (10/29)	69 (20/29)	69 (20/29)	52 (15/29)	45 (13/29)	35 (10/29)	45 (13/29)
Nasal CS	44 (11/25)	28 (7/25)	28 (7/25)	80 (20/25)	56 (14/25)	52 (13/25)	44 (11/25)	40 (10/25)	48 (10/25)
Nasal AH (Astelin)	83 (5/6)	67 (4/6)	67 (4/6)	83 (5/6)	100 (6/6)	67 (4/6)	83 (5/6)	67 (4/6)	100 (6/6)

AH, H1 antagonists; CS, corticosteroids.

**Table 3:** Questionnaire Responses by Patients in a Seasonal Allergic Rhinitis Trial Investigating MP29-02

	MP29-02	Azelastine	Fluticasone	Placebo
Triggers	(N = 207)	(N = 208)	(N = 207)	(N = 209)
Temp/weather changes	143 (69%)	140 (67%)	141 (68%)	141 (68%)
Smoke	134 (65%)	133 (64%)	129 (62%)	131 (63%)
Perfumes/fragrances	127 (61%)	114 (55%)	111 (54%)	112 (54%)
Incense/candles	88 (43%)	73 (35%)	77 (37%)	87 (42%)
Cleaning products	85 (41%)	76 (37%)	77 (37%)	81 (39%)
*Any nonallergic triggers*	*183 (88%)*	*184 (89%)*	*185 (89%)*	*185 (89%)*

**Table 4:** Questionnaire Responses by Patients Enrolled in a Seasonal Allergic Rhinitis Trial Investigating MP03-33

	Azelastine	MP03-33	Placebo
Trigger	(N = 274)	(n = 285)	(n = 275)
Temp/weather changes	198 (72%)	202 (71%)	181 (66%)
Smoke	171 (62%)	175 (61%)	167 (61%)
Perfumes/fragrances	154 (56%)	159 (56%)	144 (52%)
Incense/candles	84 (31%)	106 (37%)	87 (32%)
Household/cleaning products	107 (39%)	116 (41%)	105 (38%)
Any nonallergic triggers	240 (88%)	252 (88%)	247 (89%)

### Can medication response differentiate between chronic rhinitis subtypes?

A subanalysis of the Texas cedar SAR study attempted to determine if response to previous rhinitis treatments could be useful as a phenotypic marker for further differentiating rhinitis subtypes [5]. Before study enrollment, 84% of patients had previously tried oral antihistamines, 67% nasal corticosteroid sprays, and 35% intranasal antihistamines (Astelin).

There were no statistically significant differences in satisfactory rates {[moderate + big effect]/total of treated patients} between these medications: 76% indicated that oral antihistamines had a moderate-to-big effect, 85% that nasal corticosteroids had a moderate-to-big effect, and 83% that intranasal antihistamines (Astelin) had a moderate-to-big effect (*P *= 0.2) [5]. Of interest, patients exhibiting "big effects" in response to intranasal antihistamines indicated extreme satisfaction with the medication and were more likely to manifest symptoms induced by perennial allergens (furry pets, cats, and dogs) and nonallergic irritant triggers (Table 2) [5]. These preliminary data warrant further investigation to determine whether response to medication may be a useful clinical criterion for distinguishing between CR subtypes.

## Development of an irritant index scale instrument to objectively differentiate between allergic and nonallergic rhinitis subtypes

**Figure 1:**
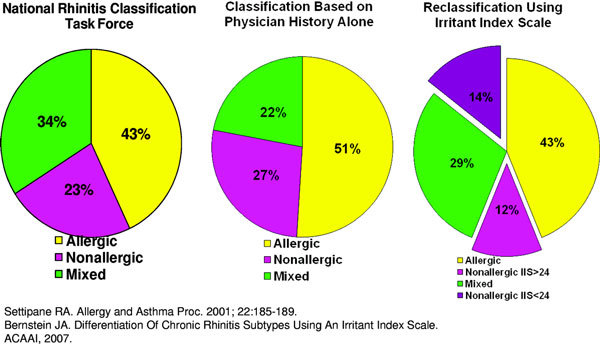
**Rhinitis subtypes: prevalence after reclassification using the Irritant Index score **[**6**,**8**].

To address the lack of an objective method for differentiating a pure AR and NAR phenotype, we recently developed an Irritant Index Scale (Appendix) to be used as an instrument for classifying CR subtypes. This instrument was initially administered to 445 patients between ages 17 and 65 years in a large clinical allergy practice, with an allergist's diagnosis of CR based on accepted criteria. A physician diagnosis of AR, which included SAR and perennial AR (PAR), required that patients had at least 1 positive skin prick test (SPT) that correlated with their clinical symptoms; a diagnosis of NAR required that patients had negative SPT with symptoms in response to odorants/chemical irritants or weather changes [6]. Nasal smears for eosinophilia to differentiate VMR from NARES were obtained. However, because both groups historically had irritant-induced symptoms, for simplicity, they were all considered NAR. Finally, MR patients were defined as having features of both AR and NAR [2,6]. All patients at the time of their office visit were administered the Irritant Index Scale, which asked them to rate the severity of their upper respiratory symptoms in response to 21 irritant triggers typically associated with NAR using a 10-point Likert scale. Triggers included ammonia, antiperspirants, bleach, cold air, cooking/frying odors, cosmetics, crude oils, fresh newsprint, hairspray, smog, cleaning products, mildew odor, paint, perfume, pine odor, soap powder, solvent, varnish, weather changes, tobacco smoke, and wood smoke. Initially, the total Irritant Index value was obtained by simply adding each trigger score and calculating the percentage of the total score (210). The original physician diagnoses of chronic rhinitis subtypes based on physician history, physical examination, and skin prick testing to a panel of aeroallergens was AR in 228 patients, NAR in 119 patients, and MR in 98 patients, respectively. Within this population, 67% were female and 90% were white. The median Irritant Index rating based on 21 triggers was 18 for AR [range 0-166], 23 for MR [range 0-190], and 24 for NAR [range 0-195]. On the basis of these findings, a cutoff value ≥ 24 was chosen to differentiate AR from NAR because it was felt that the median Irritant Index score for NAR would adequately differentiate these CR phenotypes [6]. With use of this value, patients were reclassified as follows: AR in 190 patients, NAR in 55 patients, and MR in 128 patients, respectively. Thus, use of an objective instrument for quantifying irritant-induced symptoms resulted in reclassification of 41 patients [6]. Interestingly, reclassification using this approach resulted in a prevalence of AR, NAR, and MR, very similar to what was previously reported by the National Rhinitis Classification Task Force (Figure 1) [6-8]. It is important to note that 62 NAR patients were found to have Irritant Index scores < 24, indicating that irritant-induced symptoms are not a clinical characteristic that can be uniformly used for all NAR patients [6]. However, on the basis of these preliminary findings, the Irritant Index Scale may allow for a more accurate classification of AR, NAR, and MR phenotypes based on currently accepted classical definitions of these rhinitis subtypes.

More recently, evaluation of the Irritant Index instrument has been expanded to a larger patient population and the results were reanalyzed to account for possible age and gender differences [9]. The ability to discriminate between rhinitis diagnostic categories, AR and NAR, was investigated for 153 males (AR = 124, NAR = 29) and 274 females (AR = 168, NAR = 106). Gender differences were observed in preliminary analyses; therefore, males and females responding to the Irritant Index Scale have different cut off values and should be analyzed separately [9]. Work is ongoing to identify specific Irritant Index cut off values that can accurately differentiate between allergic and nonallergic rhinitis subtypes.

## Conclusions

In summary, the Irritant Index Scale can be used to clearly differentiate between pure AR and NAR phenotypes. Patients not meeting criteria for either of these diagnoses would, by default, be classified as having MR. The subgroup of NAR patients without irritant-induced triggers requires further investigation to determine whether these individuals represent a separate, yet unrecognized, CR phenotype (ie, NAR without irritant triggers). However, development of standardized and validated questionnaires and instruments that objectively characterize chronic rhinitis subtypes appears to improve the accuracy of clinically diagnosing these patients. Using these objective criteria to establish pure rhinitis phenotypes should also improve our ability to investigate the underlying mechanism(s) of NAR, which will subsequently lead to more disease-specific treatments for this condition.

## Appendix: cincinnati irritant index scale

**Instructions: **Please rate on a scale of 0 to 10 the degree to which the following irritants cause or aggravate any upper respiratory symptoms or headaches.

"0" means that the irritant has no effect on creating or aggravating upper respiratory symptoms or headache, and "10" means that the irritant has a maximal effect. If it does not provoke the disease at all, write "0".

If you avoid the irritant because it aggravates your symptoms, please rate what your reaction was when you were exposed to the irritant in the past.

Upper respiratory symptoms may include the following: stuffy nose; runny nose; itching of the nose; sneezing; itchy, red, watery eyes; postnasal drainage.

Irritants Upper Respiratory Symptoms

1. Perfume

2. Hair spray

3. Cosmetics (including aftershave lotion)

4. Antiperspirants/deodorants

5. Fresh newsprint

6. Cooking/frying odors

7. Bleach (Clorox)

8. Soap powders (ie, laundry soap)

9. Ammonia (i.e., Lysol, Windex)

10. Household cleaners (ie, Tilex, Comet)

11. Christmas tree odors or Pine-Sol

12. Varnish

13. Solvents (turpentine, alcohol, nail polish remover)

14. Paints

15. Sawdust

16. Crude oil (gasoline, diesel, kerosene)

17. Periods of high air pollution

18. Cold air

19. Weather (rain, dampness, temperature changes)

20. Tobacco smoke/wood smoke (burning logs)

21. Mold/mildew odors

## Note

Received grant/research support from Merck, AstraZeneca, Teva Pharmaceuticals, Meda Pharmaceuticals, and Sepracor, among other companies.

Presented at a roundtable conference held in December 2008 in Washington, DC. The meeting was sponsored by the TREAT Foundation (Washington, DC) and supported through an unrestricted educational grant from Meda Pharmaceuticals. The funding company did not have any input into the development of the meeting or the proceedings, and the company was not represented at the roundtable meeting.
